# Quality of Lymph Node Dissection in Lung Cancer Surgery: A Comparative Analysis of Robotic‐Assisted Versus Video‐Assisted Thoracic Surgery Using Novel Pathological Criteria

**DOI:** 10.1002/rcs.70112

**Published:** 2025-10-11

**Authors:** Adrien Deceuninck, Pierre‐Alain Thiebaut, Michael Bubenheim, Sonia Aguir, Benjamin Bottet, Antoine Dujon, Mathias Couralet, Jean Melki, Matthieu Sarsam, Jean‐Christophe Sabourin, Florian Guisier, Jean‐Marc Baste, Nicolas Piton

**Affiliations:** ^1^ Department of Pathology UNIROUEN INSERM U1245 CHU Rouen Normandy University Rouen France; ^2^ Rouen University Hospital DRCI Rouen France; ^3^ Department of General and Thoracic Surgery Hospital Center University de Rouen Rouen France; ^4^ Department of Pneumology Rouen University Hospital Rouen France

**Keywords:** lung cancer, lymph node dissection, robot‐assisted thoracic surgery (RATS), thoracic surgery, video‐assisted surgery, video‐assisted thoracic surgery (VATS)

## Abstract

**Background:**

Lymph node dissection is essential for lung cancer staging and treatment planning. This study compares the extent and quality of lymph node dissection between robot‐assisted thoracic surgery (RATS) and video‐assisted thoracic surgery (VATS).

**Methods:**

In this prospective cohort study, 40 patients undergoing oncologic lobectomy via RATS (*n* = 20) or VATS (*n* = 20) were included. We assessed the number of explored lymph node stations, dissected nodes, and microscopic integrity criteria.

**Results:**

RATS resulted in a higher median number of explored stations (5 vs. 4; *p* = 0.0375) and resected nodes (8 vs. 6; *p* = 0.0432). However, no significant differences were found in the microscopic quality criteria.

**Conclusions:**

RATS enables broader lymph node dissection but does not improve microscopic quality compared with VATS. These findings highlight the need for further studies to assess clinical outcomes.

## 1 Introduction

Globally, lung cancer was diagnosed in approximately 2.09 million cases in 2018, making it one of the most common malignancies alongside breast cancer [1]. It is also the leading cause of cancer‐related mortality, with 1.76 million deaths reported and a 5‐year survival rate estimated at 20% [1].

Surgical resection remains the cornerstone of curative treatment for localised lung cancer [2]. Achieving an R0 resection (defined by the complete removal of the tumour with negative margins as outlined by the UICC [3]) is critical for improving patient outcomes. The International Association for the Study of Lung Cancer (IASLC) further specifies that complete resection entails:

- Removal of all identifiable tumour sites with clear resection margins (including bronchial, venous, arterial, and peribronchial soft tissues) was confirmed histologically;
- Systematic lymph node dissection;
- Absence of extracapsular tumour extension in any separately excised lymph node or those at the specimen margin; and
- A metastasis‐free highest anatomically located lymph node [4, 5].

Incomplete resections (R1 or R2) are associated with markedly poorer prognoses, with 5‐year survival rates of 36% and 28%, respectively, compared to 73% for R0 resections [6].

**FIGURE 1:**
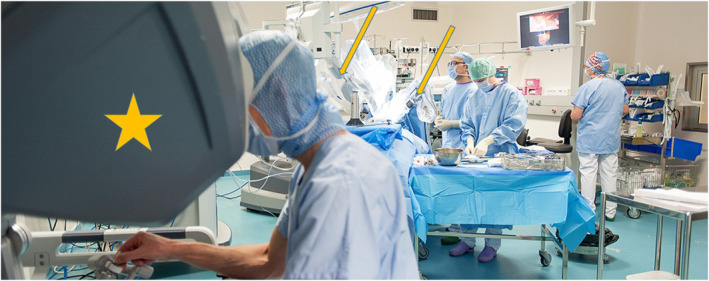
Photograph of a surgeon seated at the robotic console (indicated by the arrow) during a procedure, with the robotic instrument arms (marked by asterisks) visible in the background. Image from CHU Rouen Normandie.

Historically, lobectomy via open thoracotomy has been the standard approach for lung cancer surgery. However, over recent decades, minimally invasive techniques ‐ specifically video‐assisted thoracic surgery (VATS) and robot‐assisted thoracic surgery (RATS)—have emerged as alternatives that offer reduced postoperative pain, morbidity, mortality, and shorter hospital stays [7, 8]. VATS typically involves the insertion of one to three trocars with manual instrument manipulation, whereas RATS utilises five incisions to accommodate robotic arms and a high‐definition three‐dimensional camera, thereby providing enhanced dexterity and superior visualisation (Figure 1).

Although minimally invasive approaches have become the preferred method for stage I lung cancer surgeries [9], there remains no definitive consensus regarding the long‐term outcomes of VATS versus RATS [10, 11]. In addition to tumour resection, systematic lymph node dissection is essential for accurate staging and subsequent adjuvant therapy planning. International guidelines recommend the removal of at least 6 lymph node stations (including three mediastinal and 3 hilar/intrapulmonary stations) to ensure a comprehensive pathological assessment [12, 13].

Despite several studies comparing lymph node yield between RATS and VATS [14, 15], the literature has yet to definitively establish the superiority of one technique over the other. For instance, Huang et al. [16] demonstrated that RATS facilitates the dissection of approximately one additional lymph node station on average compared to VATS, with detection rates of occult metastases similar to those observed with open thoracotomy [17, 18]. Moreover, extracapsular extension (a factor that may influence the need for adjuvant therapy) has been highlighted as clinically significant [19].

To assess the quality of lymph node dissection, we defined three criteria:

- The number of lymph node stations explored;
- The number of lymph nodes resected; and
- The integrity of the resected lymph nodes.

**FIGURE 2:**
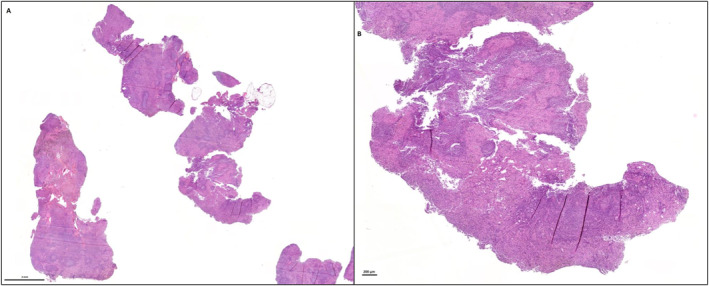
Photographs from the Pathology department of Rouen University Hospital. Haematoxylin‐eosin‐saffron‐stained histological sections of a fragmented lymph node, in which the fragments are not surrounded by a connective tissue capsule, at scales of 2 mm (A) and 200 μm (B).

**FIGURE 3:**
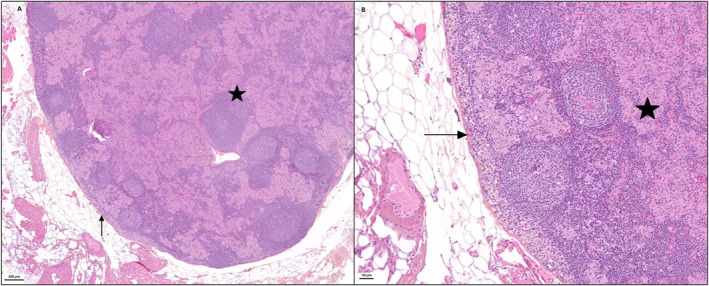
Photographs from the Pathology department of Rouen University Hospital. Haematoxylin‐eosin‐saffron‐stained histological sections (A and B) of an intact lymph node, in which the parenchyma (asterisk) is enclosed by a thin connective tissue capsule (arrow), at scales of 200 μm (A) and 50 μm (B).

While the first two criteria correspond to established IASLC recommendations [4, 12, 13], lymph node integrity, as measured by the degree of fragmentation (Figures 2 and 3, Videos 1, 2, 3, 4), has been less frequently quantified despite its potential clinical relevance.

**VIDEO 1:** Example of an incomplete excision of the station 7 mediastinal lymph node area during robot‐assisted lymph node dissection in a lung cancer patient, performed using the Da Vinci X robotic surgical system (Intuitive). Playback speed: x2. Edited using CapCut.

**VIDEO 2:** Example of a complete excision of the right hilar and 4R mediastinal lymph node areas during robot‐assisted lymph node dissection in a lung cancer patient, performed using the Da Vinci X robotic surgical system (Intuitive). Playback speed: x2. Edited using CapCut.

**VIDEO 3:** Example of an incomplete excision of the station 7 mediastinal lymph node area during video‐assisted lymph node dissection in a lung cancer patient. Playback speed: x1. Edited using CapCut.

**VIDEO 4:** Example of a complete excision of the right hilar station 10 lymph node areas during video‐assisted lymph node dissection in a lung cancer patient. Playback speed: x1. Edited using CapCut.

### 1.1 Objective

This study aimed to compare the quality of lymph node dissection between RATS and VATS using objective, measurable pathological criteria.

## 2 Material and Method

### 2.1 Ethics

Ethical approval was obtained from the local ethics committee (agreement *n*°E2025‐06) and according to the agreement of the tumour biobank of Rouen University Hospital (tissue sample collection *n*° DC2008‐689) by the institutional review board of Rouen University Hospital and by the French Ministry of Scientific Research.

### 2.2 Study Design and Patient Selection

This prospective, non‐randomised single‐centre cohort study was conducted at Rouen University Hospital. Patients aged 18 years or older with resectable lung tumours (clinical stages I, II, or IIIA according to the 8th edition TNM classification) scheduled for pulmonary lobectomy with systematic lymph node dissection were eligible. The surgical indication was confirmed during a multidisciplinary team meeting. The surgical technique, RATS or VATS, was then chosen only according to the availability of operating theatres, and not according to the characteristics of the patients or the tumour. Patients were consecutively included between May 23, 2023, and November 21, 2023. Cases were excluded if conversion to thoracotomy occurred or if intraoperative frozen section analysis was performed, as these circumstances could affect the evaluation of lymph node integrity. Only procedures performed by surgeons proficient in both RATS and VATS were considered. Five surgeons operated daily in the centre, but only three practiced both techniques regularly, and therefore participated in the study. All procedures were performed under general anaesthesia. RATS was performed using the Da Vinci X robotic surgical system (Intuitive). In our centre, four robotic arms are used for RATS, along with an additional assistant port operated by a human assistant. For VATS, an anterior triportal approach was used, as described by the Danish team led by Hansen [20].

### 2.3 Specimen Handling and Data Collection

Surgical specimens were processed in the Pathology department according to a standardised protocol. Lymph nodes were dissected and submitted separately with clear identification of the corresponding lymph node station. All pathological evaluations were performed blinded to the surgical approach. Macroscopic measurements included:

- Volume: Determined by water displacement using a graduated cylinder (accurate to 0.1 mL).
- Weight: Measured with an electronic scale (accurate to 0.01 g).
- Dimensions: Recorded as the largest and smallest axes using a tape measure.

Microscopic analysis was performed on digitised haematoxylin–eosin–saffron (H‐E‐S) stained slides (3–5 μm thickness). The QuPath software was used to quantify the lymph node parenchymal area, the length of the intact capsule and the area affected by electrocoagulation [21]. Manual annotations were performed as required, with pixel values subsequently converted to metric units.

### 2.4 Outcome Measures

The primary outcome was the number of lymph node stations explored. Secondary outcomes included:

- The number of lymph nodes resected;
- The weight and volume of lymph node fragments;
- Dimensions (long and short axes) of the fragments;
- The macroscopic appearance of the lymph node capsule (intact vs. breached);
- The size of capsular breaches;
- The microscopic analysable lymph node parenchymal area;
- The percentage of intact capsule relative to the lymph node surface; and
- The percentage of tissue areas affected by electrocoagulation.

### 2.5 Statistical Analysis

We conducted a pilot study to determine the number of 40 patients required to obtain an acceptable power. Due to the skewed distribution of some continuous variables (e.g., fragment volume), medians and interquartile ranges were used. The Hodges‐Lehmann method [22] was applied to estimate median differences and their 95% confidence intervals. The Wilcoxon‐Mann‐Whitney test was used for comparisons of continuous variables, while Pearson's Chi‐square test was employed for categorical variables. A *p*‐value < 0.05 was considered statistically significant. All analyses were performed using SAS software (SAS Institute Inc., Cary, NC, USA).

## 3 Results

### 3.1 Patient Characteristics

**FIGURE 4:**
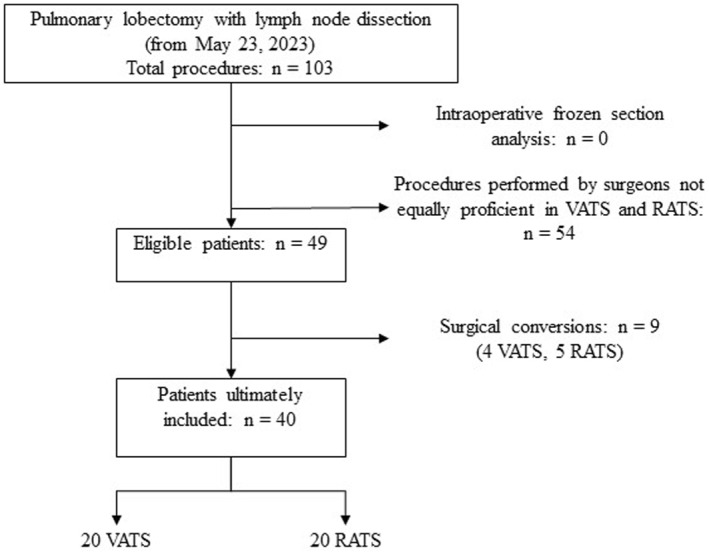
Patient inclusion diagram (RATS, Robot‐Assisted Thoracic Surgery; VATS, Video‐Assisted Thoracic Surgery).

Forty patients were included (20 RATS, 20 VATS) between May 23, 2023, and November 21, 2023 (Figure 4). Fifty‐four lobectomy cases were excluded because the operating surgeons did not equally practice both techniques at the time of inclusion. An additional 9 patients were excluded due to conversion to thoracotomy (4 in the VATS group and 5 in the RATS group), resulting in an overall conversion rate of 18.4% (16.6% for VATS and 20% for RATS; p not significant). No lymph nodes underwent frozen section analysis. Detailed patient demographics and clinical characteristics are provided in Table 1, with no significant differences between groups.

**TABLE 1:** Patient characteristics at the time of surgery.

		Total	Surgical technique		*p*‐value
			RATS	VATS	
Total	Patients	40	20	20	
Date of surgery	Min	23/05/2023	25/05/2023	23/05/2023	
	Max	21/11/2023	21/11/2023	19/10/2023	
Sex					
Male	Patients	18	9	9	1.0000
Female	Patients	22	11	11	
Age in completed years on the day of surgery	Median	65	65	62	0.9030
	Q1	60	60	60	
	Q3	71	70	71	
Smoking status					
Unknown	Patients	1	1	.	
Non‐smoker	Patients	6	4	2	0.4679
Active smoker	Patients	20	8	12	
Former smoker	Patients	13	7	6	
Clinical stage according to TNM 8th edition (cT)					
Unknown	Patients	5	1	4	
1a	Patients	1	1	.	0.1595
1b	Patients	13	6	7	
1c	Patients	6	3	3	
2a	Patients	7	6	1	
2b	Patients	1	.	1	
3	Patients	5	1	4	
4	Patients	2	2	.	
Clinical nodal stage according to TNM 8th edition (cN)					
Unknown	Patients	3	.	3	
0	Patients	34	17	17	0.0957
1	Patients	3	3	.	
Stage classification according to TNM 8th edition					
Unknown	Patients	5	1	4	
IA	Patients	19	9	10	0.1266
IB	Patients	6	5	1	
IIA	Patients	1	.	1	
IIB	Patients	6	2	4	
IIIA	Patients	3	3	.	
Neoadjuvant treatment					
No	Patients	37	17	20	0.0717
Yes	Patients	3	3	.	
WHO performance status					
Unknown	Patients	13	6	7	
0	Patients	19	10	9	0.9006
1	Patients	8	4	4	
Surgeon					
C1	Patients	10	4	6	0.5866
C2	Patients	15	9	6	
C3	Patients	15	7	8	
Operated lung part					
Right upper lobe	Patients	13	5	8	0.2187
Right middle lobe	Patients	1	.	1	
Right lower lobe	Patients	14	10	4	
Left upper lobe	Patients	7	2	5	
Left lower lobe	Patients	5	3	2	
Tumour histology					
No tumour	Patients	3	2	1	0.4542
Adenocarcinoma	Patients	26	13	13	
Squamous cell carcinoma	Patients	7	2	5	
Other malignancy	Patients	4	3	1	
Pathological stage according to TNM 8th edition (pT)					
Not applicable	Patients	2	1	1	
0	Patients	1	1	.	0.6892
is	Patients	1	.	1	
1a	Patients	2	2	.	
1b	Patients	5	2	3	
1c	Patients	6	2	4	
2a	Patients	11	6	5	
2b	Patients	3	1	2	
3	Patients	6	3	3	
4	Patients	3	2	1	
Lymph node pathological stage according to the TNM 8th edition (pN)					
Not applicable	Patients	2	1	1	
0	Patients	31	17	14	0.1930
1	Patients	4	2	2	
2	Patients	3	.	3	
Stage with pathological data according to TNM 8th edition					
Not applicable	Patients	3	1	1	
0	Patients	1	1	1	0.1173
IA	Patients	12	6	6	
IB	Patients	10	5	5	
IIA	Patients	2	.	2	
IIB	Patients	5	5	.	
IIIA	Patients	5	2	3	
IIIB	Patients	2	.	2	

*Note:* Patients indicated as ‘not applicable’ to the TNM pathological stage are those whose lung resection specimens did not contain any tumour lesions.

Abbreviations: Q1, first quartile; Q3, third quartile; RATS, robot‐assisted thoracic surgery; VATS, video‐assisted thoracic surgery.

### 3.2 Lymph Node Station Exploration and Yield

The distribution of explored lymph node stations was similar between the groups (Table 2), with station No. 7 examined in all but one VATS cases. The RATS group exhibited a significantly higher median number of explored stations (5 vs. 4; *p* = 0.0375) and a greater number of dissected lymph nodes (median 8 vs. 6; *p* = 0.0432) compared with the VATS group (Table 3).

**TABLE 2:** Comparison of surgical techniques based on explored lymph node stations.

Lymph node station	Total	Surgical technique		*p*‐value
		RATS	VATS	
Total	178	96	82	
Station no. 2R right upper paratracheal	7	3	4	0.2258
Station no. 4R right lower paratracheal	25	13	12	
Station no. 5 subaortic	10	3	7	
Station no. 6 para‐aortic	3	3	0	
Station no. 7 subcarinal	39	20	19	
Station no. 8 paraesophageal	6	6	0	
Station no. 9 pulmonary ligament	17	11	6	
Station no. 10 hilar	31	16	15	
Station no. 11 interlobar	32	16	16	
Station no. 12 lobar	8	5	3	

*Note:* The median number of explored lymph node stations was significantly higher in the RATS group compared to the VATS group (5 vs. 4, *p* = 0.0375) (Table 3), as well as the median number of dissected lymph nodes (8 vs. 6, *p* = 0.0432).

Abbreviations: RATS, robot‐assisted thoracic surgery; VATS, video‐assisted thoracic surgery.

**TABLE 3:** Comparison of surgical techniques based on the number of lymph node stations explored and the number of lymph nodes dissected.

		Total	Surgical technique		*p*‐value
			RATS	VATS	
Total	Patients	40	20	20	
Total number of lymph node stations explored	Median	4	5	4	**0.0375**
	Q1	4	4	3	
	Q3	4	6	4	
Total number of lymph nodes dissected	Median	6	8	6	**0.0432**
	Q1	4	5	4	
	Q3	8	9	7	

*Note:* Bold *p*‐values are significant (< 0.005).

Abbreviations: Q1, first quartile; Q3, third quartile; RATS, robot‐assisted thoracic surgery; VATS, video‐assisted thoracic surgery.

**TABLE 4:** Characteristics of ‘N1’ lymph nodes.

		Total	Technique chirurgicale		Median shift (VATS‐RATS) with 95% CI	*p*‐value
			RATS	VATS		
Total	Patients	38	20	18		
Total number of N1 lymph nodes resected	Median	3	3	2	−1 [−2; 0]	0.0633
	Q1	2	2	2		
	Q3	5	6	3		
Sum of fragment weights (g)	Median	0.26	0.26	0.29	0.02 [−0.10 ; 0.17]	0.7477
	Q1	0.18	0.19	0.15		
	Q3	0.41	0.37	0.45		
Sum of fragment volumes (mL)	Median	0.25	0.25	0.32	0.05 [‐0.06 ; 0.18]	0.4290
	Q1	0.20	0.18	0.20		
	Q3	0.40	0.35	0.50		
Macroscopic aspect of the lymph node capsule						
Number of patients with intact capsule	Patients	6	3	3		0.8881
Number of patients with at least one capsular breach	Patients	32	17	15		
Total number of fragments per lymph node sampled	Median	1.00	1.00	1.00	0.00 [−0.20 ; 0.19]	0.9839
	Q1	1.00	1.00	1.00		
	Q3	1.33	1.33	1.33		
Sum of the macroscopic sizes (long axis) of capsular breaches per lymph node sampled (mm)	Median	6.00	6.50	5.00	−1.63 [−3.42 ; 0.67]	0.1356
	Q1	4.33	5.53	3.67		
	Q3	7.75	7.75	8.67		
Total sum of lymph nodes surfaces on microscopic examination (mm^2^)	Median	47.21	47.21	38.37	−2.55 [−24.89 ; 29.78]	0.8151
	Q1	22.76	23.91	19.52		
	Q3	79.90	69.46	86.63		
Percentage of intact capsule surrounding the lymph nodes fragments	Median	0.90	0.86	0.91	0.04 [‐0.01 ; 0.11]	0.1069
	Q1	0.84	0.79	0.87		
	Q3	0.96	0.93	0.98		
Percentage of electrocoagulated surface of lymph nodes fragments	Median	0.03	0.03	0.04	0.00 [‐0.03 ; 0.02]	0.8596
	Q1	0.02	0.02	0.02		
	Q3	0.08	0.07	0.08		

Abbreviations: CI: confidence interval; Q1, first quartile; Q3, third quartile; RATS, robot‐assisted thoracic surgery; VATS, video‐assisted thoracic surgery.

**TABLE 5:** Characteristics of ‘N2’ lymph nodes.

		Total	Technique chirurgicale		Median shift (VATS‐RATS) with 95% CI	*p*‐value
			RATS	VATS		
Total	Patients	40	20	20		
Total number of N2 lymph nodes resected	Median	3	3	3	0 [−1; 1]	0.5131
	Q1	3	3	2		
	Q3	4	5	4		
Sum of fragment weights (g)	Median	0.54	0.64	0.47	−0.10 [−0.62; 0.14]	0.5338
	Q1	0.36	0.33	0.41		
	Q3	1.13	1.39	0.72		
Sum of fragment volumes (mL)	Median	0.56	0.66	0.53	−0.10 [−0.60; 0.14]	0.5977
	Q1	0.33	0.30	0.37		
	Q3	1.10	1.27	0.75		
Macroscopic aspect of the lymph node capsule						
Number of patients with intact capsule	Patients	4	2	2		1.0000
Number of patients with at least one capsular breach	Patients	36	18	18		
Total number of fragments per lymph node sampled	Median	1.00	1.00	1.00	0.00 [−0.25; 0.25]	0.8814
	Q1	1.00	1.00	1.00		
	Q3	1.50	1.67	1.50		
Sum of the macroscopic sizes (long axis) of capsular breaches per lymph node sampled (mm)	Median	6.20	5.50	9.00	0.61 [−2.95; 6.00]	0.8124
	Q1	4.00	4.50	4.00		
	Q3	14.00	15.40	14.00		
Total sum of lymph nodes surfaces on microscopic examination (mm^2^)	Median	60.72	66.31	54.67	−12.56 [−55.19 ; 17.38]	0.2340
	Q1	41.93	49.82	35.92		
	Q3	111.64	139.60	97.62		
Percentage of intact capsule surrounding the lymph nodes fragments	Median	0.90	0.90	0.90	−0.01 [‐0.05 ; 0.03]	0.7039
	Q1	0.88	0.89	0.88		
	Q3	0.95	0.94	0.95		
Percentage of electrocoagulated surface of lymph nodes fragments	Median	0.02	0.02	0.02	0.01 [‐0.01 ; 0.02]	0.3365
	Q1	0.01	0.01	0.01		
	Q3	0.05	0.03	0.05		

Abbreviations: CI: confidence interval; Q1, first quartile; Q3, third quartile; RATS, robot‐assisted thoracic surgery; VATS, video‐assisted thoracic surgery.

**TABLE 6:** Characteristics of lymph nodes in station 7.

		Total	Technique chirurgicale		Median shift (VATS‐RATS) with 95% CI	*p*‐value
			RATS	VATS		
Total	Patients	39	20	19		
Dimensions of lymph node fragments (short axe, mm)	Median	5	5	4	−2 [−4; 0]	**0.0285**
	Q1	3	3	3		
	Q3	7	10	6		
Dimensions of lymph node fragments (long axe, mm)	Median	17	18	17	−2 [−8; 3]	0.2418
	Q1	12	12	12		
	Q3	22	23	22		
Sum of fragment weights (g)	Median	0.57	0.64	0.43	−0.30 [−1.18; 0.02]	0.0560
	Q1	0.36	0.44	0.31		
	Q3	1.62	2.01	0.84		
Sum of fragment volumes (mL)	Median	0.50	0.70	0.42	−0.30 [−1.25; 0.03]	0.0787
	Q1	0.30	0.40	0.27		
	Q3	1.40	1.80	0.70		
Macroscopic aspect of the lymph node capsule						
Number of patients with intact capsule	Patients	10	6	4		0.5224
Number of patients with at least one capsular breach	Patients	29	14	15		
Total number of fragments per lymph node sampled	Median	1	1	1	0.00 [−0.50; 0.00]	0.7639
	Q1	1	1	1		
	Q3	2	2	2		
Sum of the macroscopic sizes (long axis) of capsular breaches per lymph node sampled (mm)	Median	10	8	11	0.00 [−8.50; 5.67]	1.0000
	Q1	6	6	4		
	Q3	19	16	19		
Total sum of lymph nodes surfaces on microscopic examination (mm^2^)	Median	88.79	107.67	51.01	−48.15 [−123.40; −1.29]	**0.0403**
	Q1	35.00	53.16	31.64		
	Q3	188.61	251.76	153.25		
Percentage of intact capsule surrounding the lymph nodes fragments	Median	0.93	0.92	0.94	0.00 [−0.07; 0.05]	1.0000
	Q1	0.83	0.87	0.80		
	Q3	0.99	0.96	1.00		
Percentage of electrocoagulated surface of lymph nodes fragments	Median	0.02	0.01	0.02	0.00 [‐0.01; 0.02]	0.5106
	Q1	0.00	0.00	0.00		
	Q3	0.05	0.03	0.07		

*Note:* Bold *p*‐values are significant (< 0.005).

Abbreviations: CI: confidence interval; Q1, first quartile; Q3, third quartile; RATS, robot‐assisted thoracic surgery; VATS, video‐assisted thoracic surgery.

Subgroup analyses (N1, N2, and station 7)

- N1 Lymph Nodes (Stations 10, 11, 12): No significant differences were observed in the number of resected nodes, fragment weight, volume, or both macroscopic and microscopic integrity parameters between groups (Table 4). One VATS patient presented with a metastatic N1 node.
- N2 Lymph Nodes (Mediastinal Stations): As shown in Table 5, the RATS and VATS groups did not differ significantly regarding the number of resected nodes or other quantitative criteria. Two metastatic N2 nodes were identified in the VATS group.
- Station 7: Analysis of lymph nodes in station No. 7 (Table 6) revealed that fragments in the VATS group had a significantly smaller short‐axis dimension (median 4 vs. 5 mm; *p* = 0.0285) and a reduced analysable parenchymal area (median 51.01 vs. 107.67 mm^2^; *p* = 0.0403) compared to RATS, although other parameters did not differ significantly.

## 4 Discussion

This study compared the quality of lymph node dissection between RATS and VATS using both quantitative macroscopic and microscopic criteria. Our findings indicate that while RATS enables a broader dissection, evidenced by a higher number of explored lymph node stations and a greater number of dissected nodes, both techniques yield comparable results regarding the microscopic quality of the dissection. Specifically, measures such as fragment volume, capsule integrity, and the proportion of intact capsules did not significantly differ between groups.

### 4.1 Interpretation and Implications

The enhanced technical precision of robotic surgery appears to facilitate more extensive lymph node clearance. However, the absence of significant differences in microscopic parameters suggests that lymph node integrity is not markedly influenced using the surgical technique.

However, this result may be influenced not only by the technical advantages of the robotic platform but also by surgeon‐related factors. Individual experience, skill level, and intraoperative decision‐making may play a key role in the extent of lymphadenectomy, regardless of the surgical approach. This should be acknowledged as a limitation and encourages future studies to control for this potential confounding factor, either through multivariate analyses adjusting for the ‘surgeon’ variable, or through multicenter designs with harmonised lymphadenectomy protocols.

Moreover, it has been reported that robotic‐assisted surgery allows surgeons to replicate open surgical gestures more easily, making it easier to perform a planned dissection compared to VATS [23], especially for the average experienced surgeon (excluding highly skilled VATS experts). This raises the question of whether, during a surgeon's learning curve, lymphadenectomy could be more effective when performed using RATS rather than VATS [16, 23].

### 4.2 Comparison With the Literature

Our results align with previous studies reporting a higher lymph node yield with RATS compared with VATS [16, 24]. Additional station exploration may potentially translate into improved staging accuracy; however, further research is needed to confirm whether this technical advantage impacts long‐term patient outcomes.

These findings are consistent with the prospective study by Novelli et al. [25], which compared RATS, VATS, and open surgery in 169 patients undergoing anatomical lung resections. In the matched RATS and VATS groups, RATS was associated with a significantly higher number of hilar lymph nodes and nodal stations removed (8 vs. 5, *p* = 0.01 and 5 vs. 4, *p* < 0.0001, respectively).

A recent propensity‐matched study by Lampridis et al. [26] compared RATS and VATS in lung cancer surgery. The study showed that RATS had a longer operative time but less blood loss than VATS. No significant differences were found in postoperative complications, length of hospital stay, or mortality. Although VATS resulted in a higher number of dissected lymph‐node stations (5.9 vs. 4.8; *p* = 0.001), the rate of pathological upstaging was similar between the groups. These data corroborate our findings regarding lymph node dissection.

Moreover, extracapsular extension, which can influence adjuvant treatment decisions, has been highlighted as clinically significant [19].

Nonetheless, it must be emphasised that oncological quality resections can also be achieved using VATS.

### 4.3 Study Limitations

Several limitations should be acknowledged. First, the monocentric design and relatively small sample size may restrict the generalisability of our findings. Second, although we included only surgeons proficient in both techniques to reduce selection bias, inter‐individual variability in surgical practices may still exist. The impact of the learning curve associated with each technique was not assessed and represents another important limitation. Third, lymph nodes attached to the lung resection specimen were not separately analysed, potentially underestimating the overall quality of the dissection. Finally, variability in the composition of excised tissue (i.e., lymph node vs. surrounding adipose tissue) may have influenced volume measurements.

### 4.4 Future Directions

Future studies should aim to include larger, multicentric cohorts and explore additional quality criteria. Moreover, specific attention should be given to the subgroup of surgeons in training or during the early stages of their learning curve, as differences between techniques might be more apparent in this population. Further research is also warranted to assess the clinical impact of more extensive lymph node dissection on long‐term outcomes such as recurrence rates and overall survival.

## 5 Conclusion

In conclusion, while RATS facilitates a more extensive lymph node dissection, with a higher number of explored stations and excised nodes, there are no significant differences between RATS and VATS regarding the microscopic quality of the resected lymph nodes. These findings suggest that the primary technical advantage of RATS lies in its ability to achieve a broader dissection rather than in enhancing lymph node integrity. RATS and VATS remain surgical tools, and ultimately, the quality of the resection depends on the surgeon's expertise and judgement. Further multicentric, prospective studies are needed to confirm these findings and elucidate their impact on clinical outcomes. In particular, investigations focusing on surgeons‐in‐training may help clarify how these technologies can support learning and improve oncological quality.

## Author Contributions

N.P., J.M.B., A.D., S.A., M.B., and B.B. designed the study. N.P., A.D., P.A.T., S.A., B.B., A.D., M.C., J.M., M.S., and J.M.B. collected the data. N.P., A.D., P.A.T., and M.B. analysed the data. N.P. and A.D. wrote the manuscript. N.P. and A.D. revised the manuscript. All authors have read and approved the final version.

## Ethics Statement

Ethical approval was obtained from the local ethics committee (agreement *n*°E2025‐06) and in accordance with the tumour biobank agreement of Rouen University Hospital (tissue sample collection *n*° DC2008‐689) by the institutional review board of Rouen University Hospital and the French Ministry of Scientific Research.

## Conflicts of Interest

The authors declare no conflicts of interest.

## Permission to Reproduce Material From Other Sources

The authors have nothing to report.

## Data Availability

The authors declare that data are available on request.
